# Application of silicalite-modified electrode for the development of sucrose biosensor with improved characteristics

**DOI:** 10.1186/s11671-015-0853-z

**Published:** 2015-03-27

**Authors:** Viktoriya M Pyeshkova, Oleksandr Y Dudchenko, Oleksandr O Soldatkin, Berna Ozansoy Kasap, Florence Lagarde, Burcu Akata Kurç, Sergei V Dzyadevych

**Affiliations:** Laboratory of Biomolecular Electronics, Institute of Molecular Biology and Genetics, National Academy of Sciences of Ukraine, 150 Zabolotnogo Str., 03680 Kyiv, Ukraine; Institute of High Technologies, Taras Shevchenko National University of Kyiv, 64 Volodymyrska Street, 01601 Kyiv, Ukraine; Micro and Nanotechnology Department, Middle East Technical University, Ankara, 06531 Turkey; Université Claude Bernard Lyon 1, Institut des Science Analytiques, 5 rue de la Doua 69100 Villeurbanne, Lyon, France

**Keywords:** Sucrose biosensor, Silicalite, Conductometric transducer

## Abstract

The application of silicalite for improvement of working characteristics of conductometric enzyme biosensors for determination of sucrose was studied in this research. Biosensors based on different types of silicalite-modified electrodes were studied and compared according to their analytical characteristics. Polyethylenimine/glutaraldehyde/silicalite-modified biosensors showed higher sensitivity compared with others type of biosensors. Moreover, the polyethylenimine/glutaraldehyde/silicalite sucrose biosensors were characterized by high selectivity and signal reproducibility (relative standard deviation (RSD) = 2.78% for glucose measurements and RSD = 3.2% for sucrose measurements). Proposed biosensors were used for determination of sucrose in different samples of beverages. The obtained results had good correlation with results obtained by HPLC. Thus, polyethylenimine/glutaraldehyde/silicalite-modified biosensors have shown perspective characteristics for the development of effective conductometric enzyme biosensors.

## Background

In order to improve immobilization process which is crucial for creating of high-performance biosensors, various techniques are used. In recent time, very often different types of nanoparticles become more popular for biosensor creation. One of these perspective nanomaterials is zeolite. Zeolites are hydrated microporous crystalline minerals. They are composed mainly of silicon, aluminum and oxygen. The modification of crystal structures makes it possible to obtain zeolites with different properties [1,2]. The regular microporous structure of the zeolite guarantees an improvement of the chemical and physical stabilities of the immobilized agent, whereas the porosity of the zeolite keeps open the access of the guest molecules or ions to the ambient. Furthermore, zeolites are able to exchange ions with some compounds. An important characteristic of zeolites is the Si/Al ratio. The increase of the Si/Al ratio results in higher thermal stability and hydrophobicity as in silicalite. Silicalite is one of the most studied zeolites that has both hydrophobic and organophilic selectivities. Also, silicalite has high thermal and chemical stabilities and high adsorption properties [3,4].

It has been previously reported on the development of biosensors based on zeolites for glucose determination [5,6]. Glucose oxidase was immobilized on the electrode surface modified with different types of zeolites by physical adsorption and by standard glutaraldehyde (GA) method. It has been found that biosensors based on zeolites had higher sensitivity comparing with others biosensors. Sensitivity and response time of developed biosensors depend on the amount of zeolite on the transducer’s surface [7].

Few modifications were reported on the immobilization procedure using silicalite and zeolite Beta for the conductometric urea biosensor creation [8]. A natural zeolite clinoptilolite was also used for the same purpose [9]. It was demonstrated that the characteristics of the conductometric urea biosensors based on urease adsorbed on silicalite are better than those of the biosensors based on urease immobilized only in GA vapor [10].

The usage of zeolites for fabrication of the biosensors for H_2_O_2_ detection based on cytochrome c [11] and the biosensor for DNA determination based on Ag/NaA zeolite-modified carbon paste electrode [12] were also considered, which appeared to be a very promising approach to further zeolites usage.

Therefore, the application of silicalite for the improvement of working characteristics of conductometric biosensors for sucrose determination was studied in this research.

## Methods

### Materials

The following enzymes and reagents were used: mutarotase (MUT) (ЕС 5.1.3.3) from pig’s kidney with activity of 100 U/mg from Biozyme Laboratories Ltd. (Blaenavon, UK); glucose oxidase (GOx) from *Penicillium vitale* (ЕС 1.1.3.4) with activity of 130 U/mg from Diagnosticum (L’viv, Ukraine); invertase (INV) (ЕС 3.2.1.26) from baker’s yeast with activity of 355 U/mg from Fluka AG - Chemische Fabrik (St. Galen, Switzerland); bovine serum albumin (BSA) (V fraction) from Sigma-Aldrich Chemie GmbH (Schnelldorf, Germany); 50% aqueous solution of GA, Sigma-Aldrich Chemie GmbH (Schnelldorf, Germany); glucose, maltose, lactose, and sucrose were from Sigma-Aldrich Chemie GmbH (Schnelldorf, Germany). Other non-organic compounds were of analytical grade.

### Conductometric transducers

The conductometric transducers were 5 × 30 mm^2^ in size and consisted of two identical pairs of stainless steel interdigitated electrodes deposited onto a ceramic support. The usage of two electrode pairs enabled differential mode of measurements.

**Figure 1:**
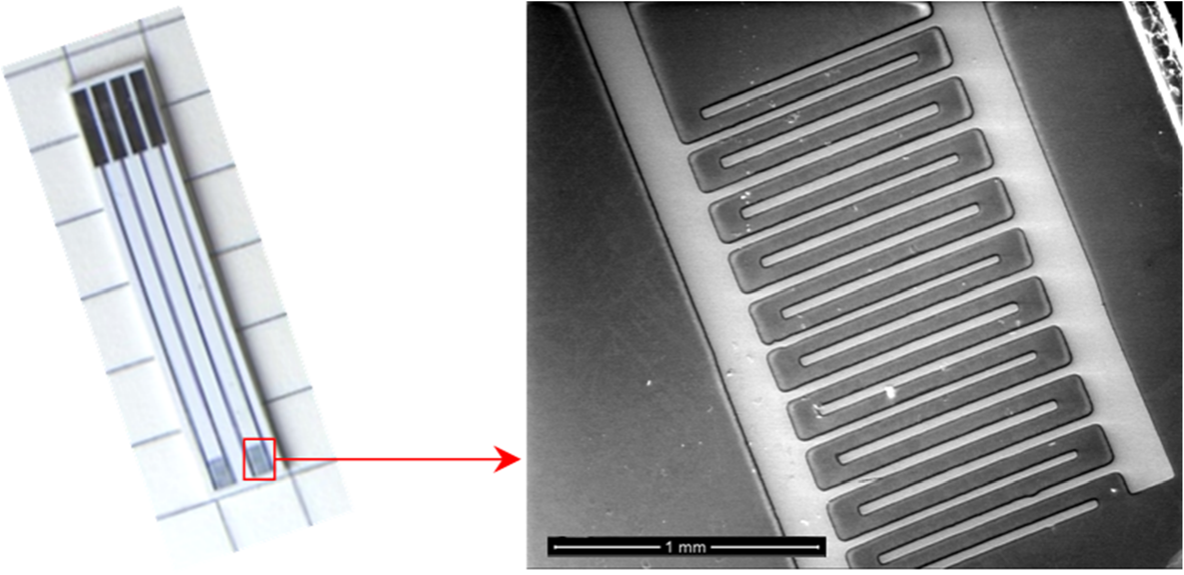
**Image of stainless steel conductometric transducer.**

The sensitive area of each electrode pair was about 1.5 × 2 mm^2^. The digits as well as interdigital spaces were 50 μm wide each. The image of stainless steel interdigitated electrodes obtained by scanning electron microscopy and the overall view of the conductometric transducer are presented in Figure 1.

### Synthesis and characterization of silicalite

**Figure 2:**
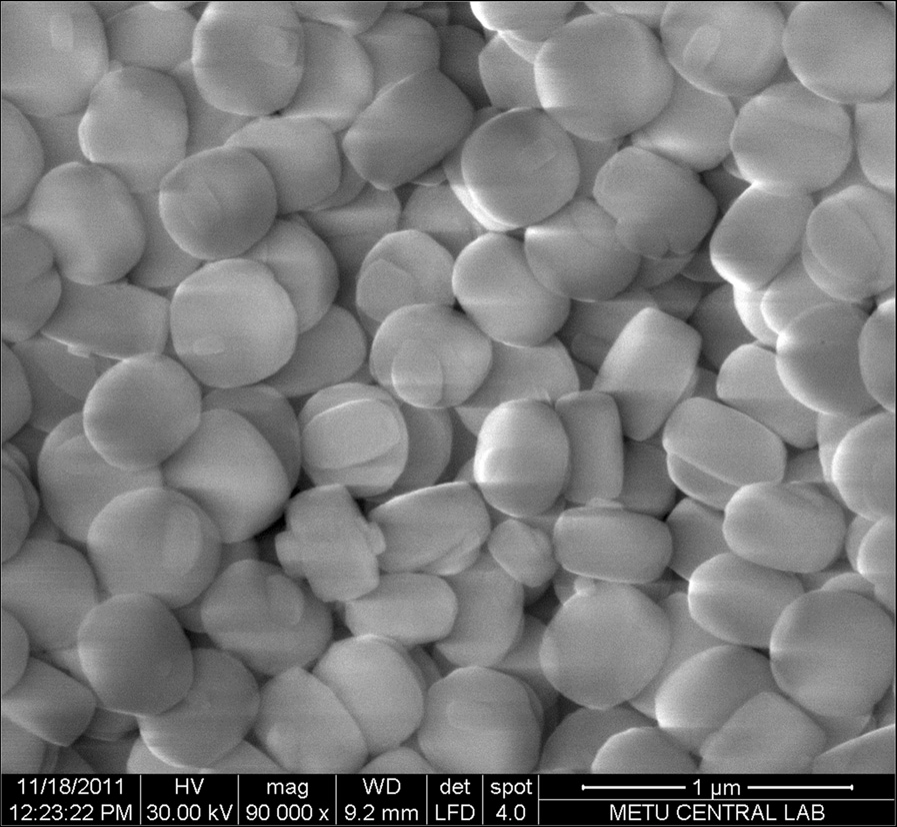
**Scanning electron microscope image of silicalite.**

**Figure 3:**
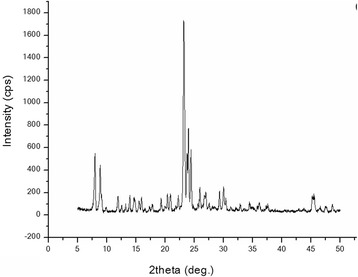
**XRD spectrum of silicalite.**

Silicalite was synthesized in the Middle East Technical University (Ankara, Turkey). The optimized molar composition of the solution used for synthesis of silicalite-1 is 1TPAOH:4TEOS:350H_2_O. Tetraethylorthosilicate (TEOS, 95%) was used as the silica source. Tetrapropylammonium hydroxide (TPAOH, 25%) was used as a template. By hydrolyzing tetraethoxysilane (TEOS) with tetrapropylammonium hydroxide (TPAOH) solution, a clear homogeneous solution was obtained at room temperature for 6 h under stirring. Afterwards, the resulting solution was placed in oven for 18 h at 125°C. Then to remove the unreacted material, the crystallized solid particles were centrifuged at 13,000 rpm, washed with deionized water, and dried at 80°C. The SEM (scanning electron microscope) image of synthesized silicalite depicted in Figure 2 shows that the prepared silicalite particles have size about 400 to 500 nm. X-ray diffraction (XRD) spectrum of silicalite is shown in Figure 3.

### Creation of PEI silicalite-modified electrode

For preparing of biosensors based on PEI silicalite-modified electrodes, the electrode surfaces were dip-coated with mucasol for 15 min, rinsed with copious amount of distilled water, and dried under air. For formation of homogeneous layers of PEI, both drop coating and spin coating techniques have been tried since spin coating gave more homogeneous layers, it was continued to be used. In our study, polyethylenimine (PEI) was used as a layer between electrode surface and silicalite. The suitable conditions for silicalite monolayer production were chosen as spin coating with 0.5% PEI in ethanol at 3,000 rpm with 15 s and 100°C for calcination temperature during 30 min. This kind of PEI silicalite-modified electrode was used as a base for the preparation of polyethylenimine/silicalite (PEI/Sil) and polyethylenimine/glutaraldehyde/silicalite (PEI/GA/Sil) biosensors.

### Creation of silicalite-modified electrode

A silicalite layer on the transducer surface was formed using drop-coating technique. We used 10% (*w*/*w*) silicalite solution in 5 mM phosphate buffer, pH 6.5. A constant amount of silicalite solution (0.2 ml) was deposited onto the active zone of each pair of electrodes, and then the transducer was heated for 2 min at 200°C. This temperature had no effect on the transducer working parameters. The procedure resulted in the formation of silicalite layer in the electrodes active zones. This kind of silicalite-modified electrode was used as a base for the preparation of Sil and GA/Sil biosensors.

### Creation of biomembrane

To create an active membrane of sucrose biosensors, 20 mM phosphate buffer solution at pH 7.0 which contains 5% (*w*/*w*) of GOx, 5% (*w*/*w*) of MUT, 5% (*w*/*w*) of INV, and 5% (*w*/*w*) of BSA was mixed in 1:1 ratio with 2% (*w*/*w*) aqueous solution of GA for the immobilization on the surface of bare or silicalite-modified electrodes.

In order to obtain an active membrane of glucose biosensors, 20 mM phosphate buffer solution at pH 7.0 which contains 10% (*w*/*w*) of GOx and 5% (*w*/*w*) of BSA was mixed in 1:1 ratio with 2% (*w*/*w*) aqueous solution of GA in the same way as described above.

The mixture for reference membrane was prepared in analogous manner, except that the enzymes were replaced with BSA. Thus, the reference solution for sucrose biosensor contained 20% (*w*/*w*) BSA totally and 15% (*w*/*w*) BSA in the case of glucose biosensor. Enzyme and reference solutions were separately mixed with 2% (*w*/*w*) aqueous solution of GA in a ratio of 1:1. Immediately afterwards, we deposited the mixture of enzyme solution with GA on one pair of electrodes and the mixture of reference solution with GA was placed on another one. The time of immobilization was 15 min; GA formed strong covalent bonds between the compounds of bioselective membrane, whereas the whole bioselective membrane was attached to the electrode surface through weak (i.e., Van der Waals) bonds. After immobilization, the electrodes were submerged in the working buffer for 15 min to wash out the unbound enzyme and GA excess.

**Figure 4:**
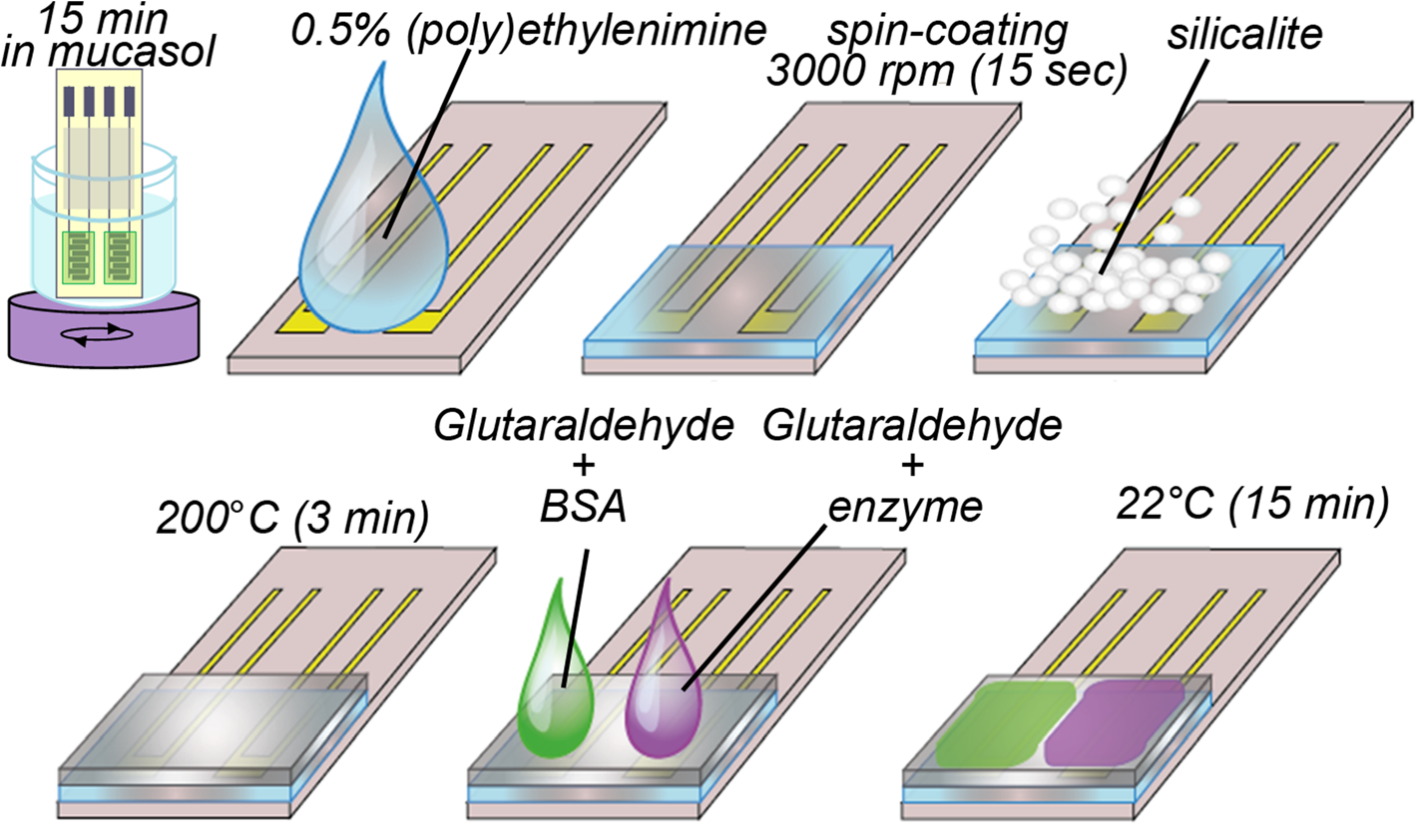
**Creation of PEI/GA/Sil biosensor.**

An example of following the procedure of creation of PEI/GA/Sil biosensor is shown in Figure 4.

### Electrochemical measuring system

A portable conductometric analyzer was used to determine changes in conductivity in the near-electrode layer. The portative measuring device (9.5 cm × 2.5 cm × 13.5 cm) was produced in the Institute of Electrodynamics of National Academy of Sciences of Ukraine (Kiev, Ukraine). The applied sinusoidal potential with frequency of 36.5 kHz and amplitude of 14 mV allowed avoiding such effects as Faraday processes, double-layer charging, and polarization of the microelectrodes. Illumination and temperature variations had practically no influence on the biosensor characteristics. The measurements were carried out in a glass cell filled with phosphate buffer (volume 1 ml), under vigorous magnetic stirring.

The conductometric determination of sucrose and glucose, using the prepared biosensors, was realized in a differential measuring mode, which ensured satisfactory detection accuracy and suppression of non-informative effects of the environment (variations of temperature, рН, and background conductivity of working solution).

### Measurement procedure

Measurements were carried out at room temperature in 5 mM phosphate buffer solution, pH 6.5, continuously stirred in an open 1 ml cell. The substrate concentrations in the cell were varied by the addition of the different volumes of the stock solution. All experiments were repeated in triplicate. The data in the figures were expressed either as a mean of three repeated results of experiment or as a mean ± standard deviation (SD). The experiments were performed at least in three series.

## Results and discussion

The cascades of enzymatic reactions for sucrose detection by conductometric biosensor are presented below:



Three enzymes are used for measurement of sucrose while only one, glucose oxidase - for glucose. The enzyme invertase decomposes sucrose into β-D-fructose and α-D-glucose, which is transformed into β-D-glucose by mutarotase. β-D-glucose is decomposed by GOD to hydrogen peroxide and D-glucolactone. In its turn, D-glucolactone is spontaneously hydrolyzed to gluconic acid, which dissociates to the acid residue and a proton. These reactions lead to changes in solution conductivity what can be registered by a conductometric transducer.

First of all, we check the sensitivity of five types of biosensors based on different types of enzyme immobilization: (1) biosensors with GA without silicalite, (2) PEI silicalite-modified biosensors without immobilization in GA (PEI/Sil biosensor), (3) PEI silicalite-modified biosensors with immobilization in GA (PEI/GA/Sil biosensor), (4) silicalite-modified biosensors without immobilization in GA (Sil biosensor), and (5) silicalite-modified biosensor with immobilization in GA (GA/Sil biosensor) (Table 1). The PEI/GA/Sil biosensors are characterized by much higher sensitivity than the biosensors without GA and biosensors based only on GA cross-linking.

**Table 1:** **Responses of biosensors based on different types of enzyme immobilization**

	**Biosensors with different type of enzyme immobilization**				
	**Biosensor with GA without silicalite**	**PEI/Sil biosensor**	**PEI/GA/Sil biosensor**	**Sil biosensor**	**GA/Sil biosensor**
Response to 0.5 mM substrate (μS)	0.47 ± 0.38	0.41 ± 0.32	14.53 ± 4.1	0.23 ± 0.18	5.04 ± 0.95

Thus, the method of enzyme immobilization with GA on PEI silicalite-modified electrodes was chosen as the most appropriate for the further biosensor fabrication; for this reason, the next study was carried out using only PEI/GA/Sil biosensors.

**Figure 5:**
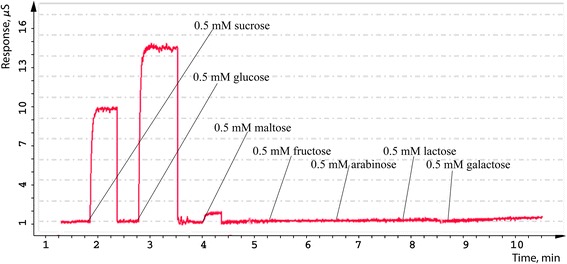
**Selectivity test of PEI/GA/Sil sucrose biosensor.** Measurements were carried out in 5 mM phosphate buffer solution.

First of all, we studied the selectivity of PEI/GA/Sil sucrose biosensors. Biosensor selectivity, one of important analytical characteristics, depends on choice of both biological recognition element and transducer. An influence of interfering components (some carbohydrates) on response value was studied to test biosensor selectivity. For that purpose, glucose, maltose, sucrose, lactose, fructose, arabinose and galactose in concentration of 0.5 mM were added to a working cell (Figure 5).

In general, conductometric biosensors appeared to be sufficiently selective. There was no significant influence of common carbohydrates except of sucrose and glucose, which is expected. The sucrose biosensors are highly sensitive not only to sucrose but also to glucose because the enzyme membrane of sucrose biosensors contains also glucose oxidase. For this reason, to determine sucrose in multicomponent solution, we used two biosensors: sucrose biosensor that gives the summary response to both substrate (sucrose and glucose) and glucose biosensor that selectively determines only glucose.

**Figure 6:**
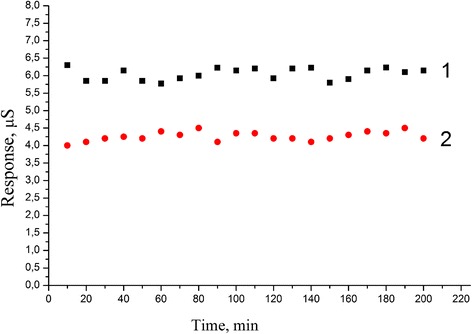
**Signal reproducibility of PEI/GA/Sil sucrose biosensor.** Responses of sucrose biosensor: (1) responses to 0.25 mM glucose, (2) responses to 0.25 mM sucrose.

Reproducibility is one of the most important working characteristics of biosensors. To determine signal reproducibility, the biosensors’ responses to 0.25 mM glucose and sucrose were measured during one working day with 10- to 15-min intervals. The biosensors were kept in the continuously stirred buffer solution at room temperature during intervals between measurements. As seen from Figure 6, the biosensor responses were highly reproducible. The relative standard deviation (RSD) for glucose measurements was 2.78% and for sucrose measurements was 3.2% which is quite acceptable value (Figure 6).

**Figure 7:**
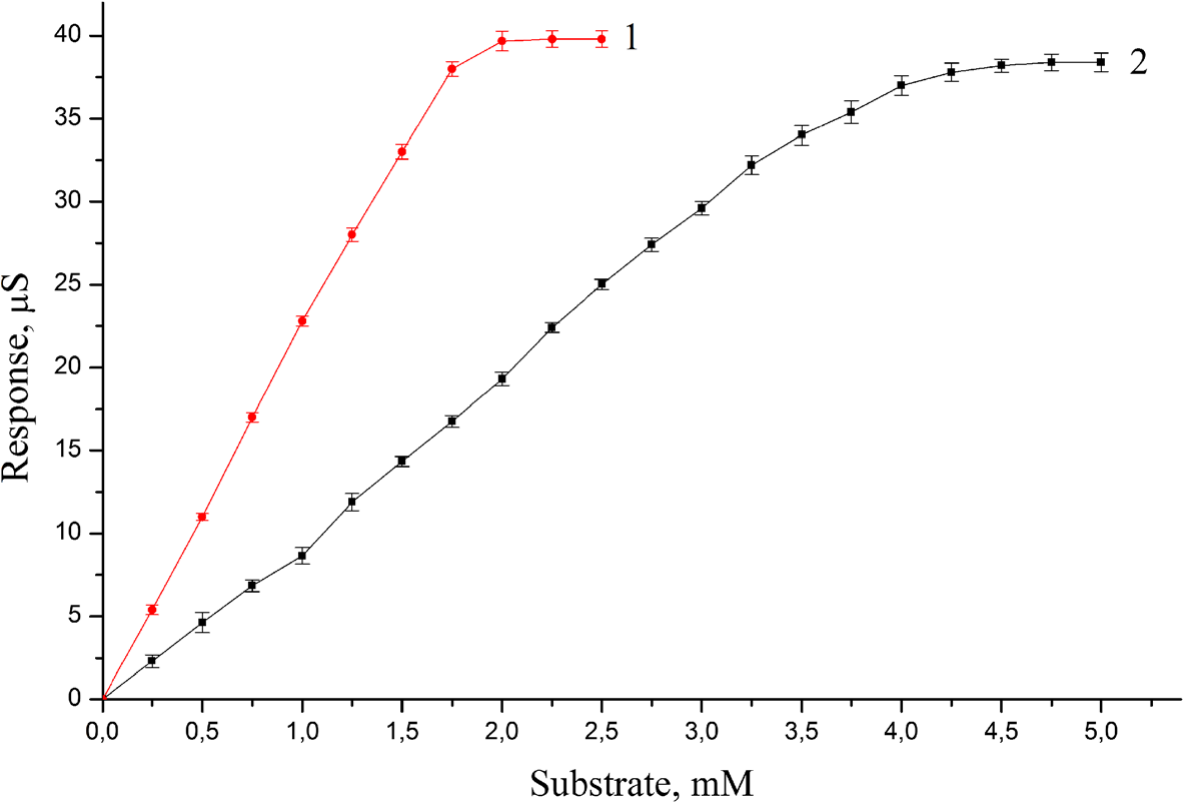
**Calibration curves of PEI/GA/Sil sucrose biosensors.** Responses are shown for adding of glucose (1) and sucrose (2) respectively.

The calibration curves to glucose and sucrose by PEI/GA/Sil sucrose biosensor are shown on Figure 7. The PEI/GA/Sil sucrose biosensors showed extended linear range of sucrose detection comparing with traditional type of biosensors based on immobilization in GA without silicalite. The linear range of PEI/GA/Sil sucrose biosensors was 0.0035 to 4 mM for sucrose and 0.0015 to 1.75 mM for glucose (Figure 7). The limit of detection (LOD) for glucose determination was 1.5 μM and LOD for sucrose determination was 3.5 μM.

We used PEI/GA/Sil biosensors for the determination of sucrose concentration in different sweet beverages. Obtained results were compared with results of high-performance liquid chromatography (HPLC) (Table 2). The correlation between the methods is high (*R* = 0.99).

**Table 2:** **Comparison of biosensor and HPLC analysis of beverages samples**

**Samples of beverages**	**Concentration of sucrose in samples, mM (** ***n*** **= 3)**	
	**Biosensor**	**HPLC**
Orange nectar ‘Dooy’, Turkey	42.3 ± 3.4	46.3 ± 1.8
Orange juice ‘Cappy’, Turkey	213.6 ± 6	241.8 ± 0.3
Apple juice ‘*Pinar*’, Turkey	41.5 ± 3.8	48.2 ± 0.4
Orange nectar ‘*Göze*’, Turkey	17.7 ± 1	17.9 ± 0.1

It was concluded that the method of enzyme adsorption on PEI silicalite-modified electrodes with GA is well suited for the biosensor manufacture.

## Conclusions

Conductometric enzyme biosensors based on different type of silicalite-modified electrodes were created and compared. PEI/GA/Sil biosensors have shown higher sensitivity to substrate compared with others biosensors tested in this work. Moreover, the PEI/GA/Sil sucrose biosensors were characterized by high selectivity and signal reproducibility (RSD = 2.78% for glucose measurements and RSD = 3.2% for sucrose measurements). PEI/GA/Sil sucrose biosensors were used to determine the concentrations of sucrose in different beverage samples. The results of biosensor measurements of sucrose in beverages had a high correlation (*R* = 0.99) with the results obtained by HPLC. Thus, the method of enzyme immobilization using PEI/GA/Sil composition is highly effective and perspective for biosensor creation.

## Abbreviations

GA: glutaraldehyde
HPLC: high-performance liquid chromatography
LOD: limit of detection
PEI: polyethylenimine
RSD: relative standard deviation
Sil: silicalite
